# Health workforce metrics pre- and post-2015: a stimulus to public policy and planning

**DOI:** 10.1186/s12960-017-0190-7

**Published:** 2017-02-15

**Authors:** Francisco Pozo-Martin, Andrea Nove, Sofia Castro Lopes, James Campbell, James Buchan, Gilles Dussault, Teena Kunjumen, Giorgio Cometto, Amani Siyam

**Affiliations:** 1 Instituto de Cooperación Social Integrare, calle Balmes 30, 3-1, 08007 Barcelona, Spain; 2 Health Systems and Innovations, WHO Headquarters, Geneva, Switzerland; 3 Global Health Workforce Network, WHO Headquarters, Geneva, Switzerland; 4 School of Nursing Midwifery and Health, University of Technology Sydney, Sydney, Australia; 5 Global Health and Tropical Medicine, Instituto de Higiene e Medicina Tropical, Universidade Nova de Lisboa, Lisbon, Portugal

**Keywords:** Health workforce, Health systems, Metrics, Countdown, Data, Densities, Low- and middle-income countries, Universal health coverage, Sustainable development goals

## Abstract

**Background:**

Evidence-based health workforce policies are essential to ensure the provision of high-quality health services and to support the attainment of universal health coverage (UHC). This paper describes the main characteristics of available health workforce data for 74 of the 75 countries identified under the ‘Countdown to 2015’ initiative as accounting for more than 95% of the world’s maternal, newborn and child deaths. It also discusses best practices in the development of health workforce metrics post-2015.

**Methods:**

Using available health workforce data from the Global Health Workforce Statistics database from the Global Health Observatory, we generated descriptive statistics to explore the current status, recent trends in the number of skilled health professionals (SHPs: physicians, nurses, midwives) per 10 000 population, and future requirements to achieve adequate levels of health care in the 74 countries. A rapid literature review was conducted to obtain an overview of the types of methods and the types of data sources used in human resources for health (HRH) studies.

**Results:**

There are large intercountry and interregional differences in the density of SHPs to progress towards UHC in Countdown countries: a median of 10.2 per 10 000 population with range 1.6 to 142 per 10 000. Substantial efforts have been made in some countries to increase the availability of SHPs as shown by a positive average exponential growth rate (AEGR) in SHPs in 51% of Countdown countries for which there are data. Many of these countries will require large investments to achieve levels of workforce availability commensurate with UHC and the health-related sustainable development goals (SDGs). The availability, quality and comparability of global health workforce metrics remain limited. Most published workforce studies are descriptive, but more sophisticated needs-based workforce planning methods are being developed.

**Conclusions:**

There is a need for high-quality, comprehensive, interoperable sources of HRH data to support all policies towards UHC and the health-related SDGs. The recent WHO-led initiative of supporting countries in the development of National Health Workforce Accounts is a very promising move towards purposive health workforce metrics post-2015. Such data will allow more countries to apply the latest methods for health workforce planning.

**Electronic supplementary material:**

The online version of this article (doi:10.1186/s12960-017-0190-7) contains supplementary material, which is available to authorized users.

## Background

The case for universal health coverage (UHC) is well-established, but its implications for the health workforce have only recently started to receive attention. Countries working towards UHC need to keep track of the size and composition of their health workforce and to anticipate future need for human resources for health (HRH) [1]. This can be strategically informed by valid and reliable workforce data [2]; without these data, decision-makers are unable to plan strategically or anticipate future needs [3, 4].

The importance of HRH data and the need to improve them has been stressed by the World Health Organization (WHO), the World Bank and the Organization for Economic Co-operation and Development [5–7]. To date, data collection processes and mechanisms have tended to be developed at a country level. There have been attempts to create harmonised regional and global data sets [8, 9], but this work requires further development.

Recent data from the International Labour Organization estimate a global shortfall of over 10 million health workers and affecting principally countries with the highest burden of mortality and morbidity [10]. The focus of this paper is on the 75 ‘Countdown to 2015’ countries. Countdown to 2015 was a global movement which tracked progress towards the health-related Millennium Development Goals (MDGs) in the 75 countries where more than 95% of maternal and child deaths occurred [11]. The Countdown to 2015 collaboration has now evolved into the ‘Countdown to 2030 for Reproductive, Maternal, Newborn, Child, and Adolescent Health and Nutrition’ initiative, which will continue the focus on high-burden countries, particularly in Sub-Saharan Africa and South Asia [12], making these 75 countries still highly relevant in the post-2015 era.

This paper uses two health worker density thresholds to assess the HRH situation in the Countdown countries. Firstly, the 2006 World Health Report [13] stated that countries with fewer than 22.8 physicians, nurses and midwives per 10 000 population were highly unlikely to be able to provide 80% coverage of the most basic health services [14]. Secondly, WHO recently developed an ‘SDG Index threshold’ as an indicative minimum density representing the need for health workers to achieve the health targets of the Sustainable Development Goals (SDGs). The value of the threshold was determined to be 4.45 doctors, nurses and midwives per 1000 population (or 44.5 per 10 000) [15].

These two thresholds are both needs-based yet vary in interpretation: countries below the 22.8 threshold may be thought of as having too few health workers to meet even the most basic health needs, whereas the 44.5 threshold can be thought of as a step forward in identifying the minimum health workforce requirements to achieve the health-related SDGs.

As stated in the 2006 World Health Report, these thresholds ‘are not a substitute for specific country assessments of sufficiency, nor do they detract from the fact that the effect of increasing the number of health workers depends crucially on other determinants’ [13]. Additionally, there are limitations in both these thresholds and the quality of the data used to calculate countries’ health worker densities [16–19]. However, in the absence of robust estimates of HRH development, thresholds offer a common comparative value against which countries can be monitored to check HRH progress or the lack of it [20]. Therefore, this study aimed to (a) describe HRH metrics in the 75 Countdown countries using a global and comparable source and (b) describe and assess some commonly used HRH metrics, their sources of data, and the methods used to analyse HRH data in the research literature. The fulfilment of these two objectives allows us to make some recommendations about how HRH metrics (and the data that feed into them) could be developed in the SDG era.

## Methods

To describe the characteristics of HRH metrics in the 75 Countdown countries, we used two indicators of workforce availability: (1) number of skilled health professionals (SHP numbers: nurses, midwives and physicians) and (2) density of skilled health professionals per 10 000 population (SHP density). We extracted data on SHP numbers from 2004 to 2014 for each of the 75 Countdown countries from the WHO Global Health Workforce Statistics database [21]. This database compiles data from four main sources: population censuses, labour force and employment surveys, health facility assessments and routine administrative information systems. Most of the data from administrative sources are derived from published national health sector reviews and/or official reports to WHO offices.

No SHP data were available for South Sudan, so this country was excluded. To calculate SHP densities for each of the remaining 74 countries, country data on SHP numbers were divided by the population size [22] of each country in the relevant year. We used descriptive statistics to examine the current levels of SHP density and their association with (1) gross domestic product (GDP) and health expenditure and (2) country-specific health outcomes and health care coverage indicators. We used Pearson’s *r* to measure the correlation between SHP density and these indicators. We conducted analysis by region by allocating each country to one of the seven UNICEF regions (the regional presentation of similar analysis presented by the Countdown initiative).

We used descriptive statistics to explore trends over time in SHP density for 53 of the 54 Countdown countries which reported SHP numbers for two points in time: 2004 (or closest year prior to 2004—the oldest data are from 1997 for Angola) and the most recent year available (see Additional file 1): Uganda was excluded from the trend analysis due to a highly discrepant change in the number of SHP reported between 2004 and 2005, the two time points available for this country). For 52 of these 53 countries, it was possible to explore trends over time disaggregated into (1) number of nurses and midwives and (2) number of physicians (the exception was Madagascar, for which such disaggregated data were not available). Trends over time were measured using the average exponential growth rate (AEGR):

1 $$ \mathrm{AEGR}=\frac{ln\left(\raisebox{1ex}{${w}_n$}\!\left/ \!\raisebox{-1ex}{${w}_1$}\right.\right)}{n}-1 $$

In Eq. 1 above, *w*_1_ and *w*_*n*_ are the first (2004 or nearest) and the latest observations of variable *w* (either SHP numbers or SHP density) in a period of *n* years. The AEGR is most suitable to define the growth rate between two points in time for certain demographic indicators, notably labour force and population. The AEGR does not correspond to the annual rate of change measured at a 1-year interval, but rather to an average rate that is representative of the available observations over the entire period.

The rationale behind exploring trends over time in both SHP numbers and SHP densities is that these two indicators measure different things: while changes in SHP numbers measure the effort made by a health care system in increasing the overall availability of skilled health professionals, changes in the SHP density measure reconciles the changes in the availability of SHP given an individual country’s population growth.

Looking forward, a final analysis calculated the AEGR required in each Countdown country to reach a density of 44.5 SHP per 10 000 population by 2030.

To complement this analysis and to inform the future development of global HRH metrics, we investigated the types of methods and data sources used in other HRH studies by means of a rapid literature review. The search was conducted in PubMed, in the *Bulletin of the World Health Organization* and in *Human Resources for Health*, using the following four search terms: ‘Human Resources for Health’, ‘Data’, ‘Metrics’ and ‘Statistics’.

The initial search yielded 1144 papers. This was narrowed down to 237 on the basis of a title review, then to 125 on the basis of an abstract review. From these, 86 were selected for full review according to the following inclusion criteria: (1) used a quantitative approach (or mixed methods including some quantitative), (2) used HRH data, (3) published in or after 2004 and (4) published in English. Only papers from peer-reviewed publications were included (i.e. there was no grey literature). Nearly 90% of the studies were published from 2007 onwards, reaching its peak in 2013 (*n* = 14). The final cutoff date for inclusion was May 2015, and the whole process was conducted by one researcher.

Relevant information about the study attributes was extracted and recorded as follows:

- The income level of the countries included in the study (high-, upper-middle, lower-middle and low-income according to the World Bank income classification)
- The type of method used (basic descriptive and inferential statistics, regression analysis, workforce modelling)
- The comparative level where the study was conducted (multi-country, national, national and subnational)
- The health workforce cadre(s) included in the analysis (physicians, nurses, midwives and others)
- The type of data sources (global, national, sub-national and administrative, institutional)
- The metrics used (headcounts, densities and others)
- The topic of interest (e.g. migration, distribution, among others)

## Results

### HRH metrics in the countdown countries

**Fig. 1:**
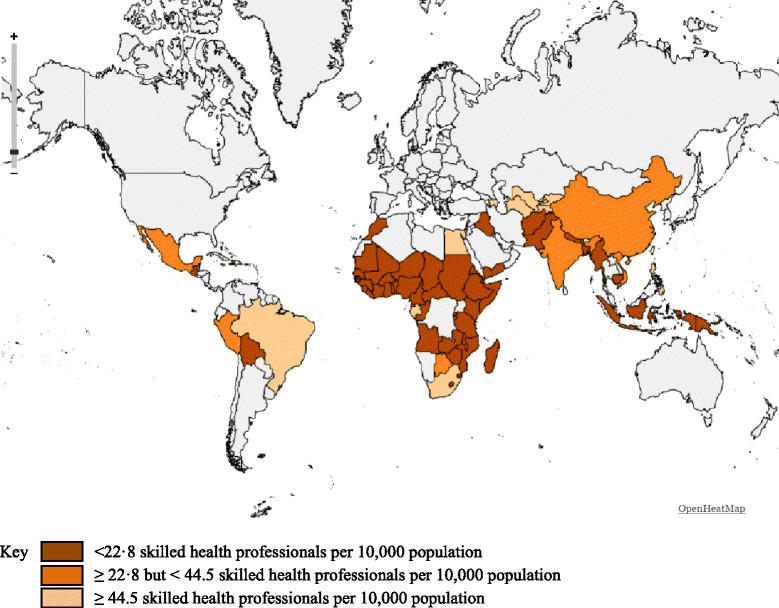
Mapping of 74 countries based on the established SHP density thresholds, most recent available year (list of countries in the Additional file 1)

Wide variations in SHP density were observed; the median SHP density in the 74 countries was 10.2 per 10 000 population, with estimates ranging from 1.6 in Madagascar and Niger to 142 in Uzbekistan. Of the 74 countries, 55 (74%) fell short of the 22.8 threshold, 8 had a SHP density between 22.8 and 44.5 and 11 had a SHP density of 44.5 or above (see Additional file 1). Most of the countries with very low densities are in sub-Saharan Africa and South Asia (see Fig. 1).

**Fig. 2:**
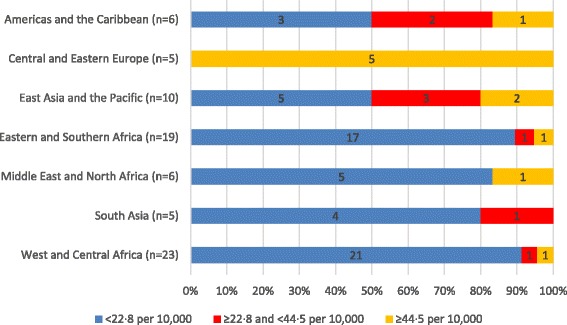
Number of physicians, nurses and midwives per 10 000 population in 74 countries, by UNICEF region

Figure 2 shows how SHP density varied by UNICEF region. All five Countdown countries in the Central and Eastern Europe region had at least 44.5 SHPs per 10 000 population. However, in other regions, SHP densities tended to be much lower.

**Fig. 3:**
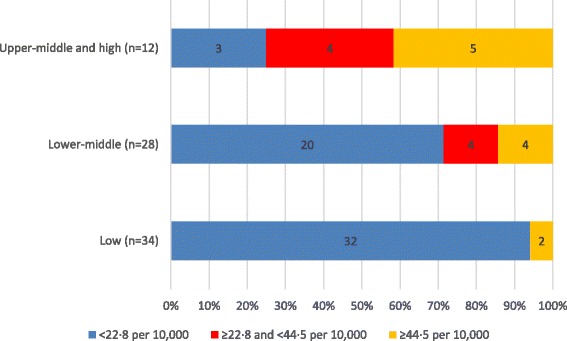
Number of physicians, nurses and midwives per 10 000 population in 74 countries, by World Bank income group

Figure 3 shows the strong association between World Bank income group [23] and SHP density: 32 (94%) of the low-income countries had a density <22.8, compared with 20 (71%) lower-middle-income countries and three (18%) upper-middle- and high-income countries.

**Fig. 4:**
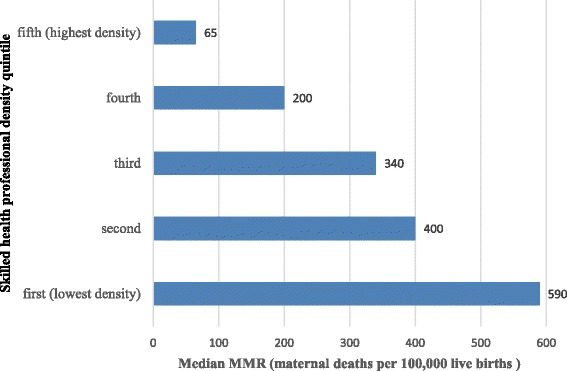
Median maternal mortality ratio (MMR), by quintiles of number of physicians, nurses and midwives per 10 000 population

**Fig. 5:**
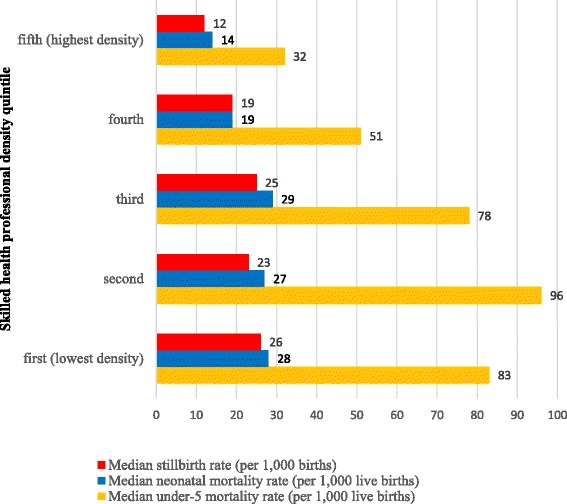
Median stillbirth, neonatal mortality and under-5 mortality rates, by quintiles of number of physicians, nurses and midwives per 10 000 population

Countries with lower SHP densities tended to have worse maternal and newborn health (MNH) outcomes, as illustrated in Figs. 4 and 5. Figure 4 shows that Countdown countries with higher SHP densities had lower maternal mortality ratios (*r* = −0.56, *p* < 0.05) according to WHO mortality estimates [24]. Countdown countries in the highest quintile of SHP density (31.2+ SHPs/10 000 population) had a median maternal mortality ratio 11% of that of countries belonging to the lowest quintile of SHP density (<4.9 SHPs/10 000 population). Figure 5 shows that countries with higher SHP densities had (1) lower stillbirth rates (*r* = −0.56, *p* < 0.05), (2) lower neonatal mortality rates (*r* = −0.45, *p* < 0.05) according to Healthy Newborn Network data [25] and (3) lower under-5 mortality rates (*r* = −0.48, *p* < 0.05) according to UN data [26]. These results cannot prove a causal relationship between health worker density and MNH outcomes, since strong confounders such as quality of care or social factors are not taken into account in this analysis. However, the contribution of health worker density to the improvement of health outcomes has been shown in other studies [27]. Moreover, it should be noted that, for most Countdown countries, estimates of the maternal mortality ratio and the stillbirth rate are generated by statistical modelling rather than empirical data [28, 29]. Factors such as GDP and gross national income (GNI) are used as predictors in the modelling, which is bound to affect the observed correlation between mortality estimates and SHP density.

**Fig. 6:**
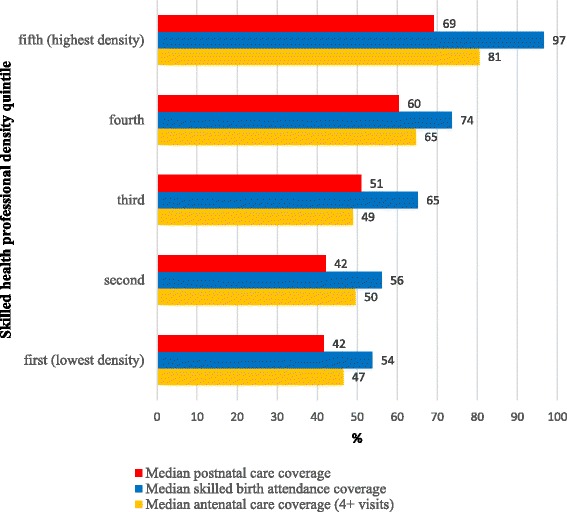
Median postnatal care coverage/median skilled birth attendance coverage/median antenatal care coverage by quintile of number of physicians, nurses and midwives per 10 000 population

Figure 6 shows that, where data are available, countries with higher SHP density had (1) higher coverage of antenatal care (*r* = 0.46, *p* < 0.05), (2) higher coverage of skilled birth attendance (*r* = 0.6, *p* < 0 · 05) and (3) higher coverage of postnatal care (*r* = 0.40, *p* < 0.05) according to Demographic and Health Surveys and UN estimates. [30, 31] Countdown countries in the highest quintile of SHP density (31.2+ SHPs/10 000 population) estimate a median skilled birth attendance coverage close to double that of those in the lowest quintile of SHP density (<4.9 SHPs/10 000 population).

**Fig. 7:**
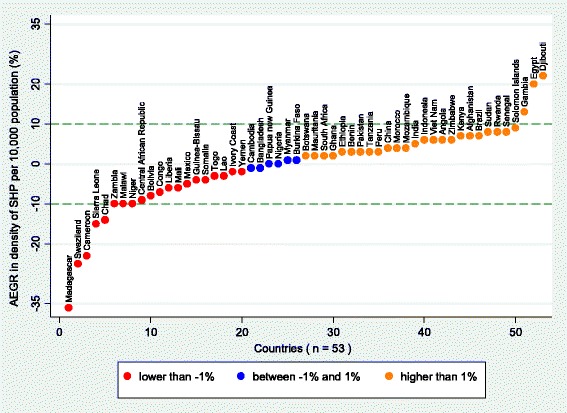
Average exponential growth rate (AEGR) in number of physicians, nurses and midwives per 10 000 population for 53 Countdown countries with more than one data point between 2004 and the latest available year

As measured by the AEGR, of the 53 countries with at least two data points, 27 (51%) showed an increase in SHP density, 19 (36%) showed a decrease and the remaining 6 (11%) showed little or no change (AEGR between −1 and 1%). Figure 7 shows which countries fall into each of these categories: Djibouti, Egypt and the Gambia all showed a positive AEGR greater than 10%. On the other hand, in Madagascar, Swaziland, Cameroon and Sierra Leone a large negative AEGR was observed.

**Fig. 8:**
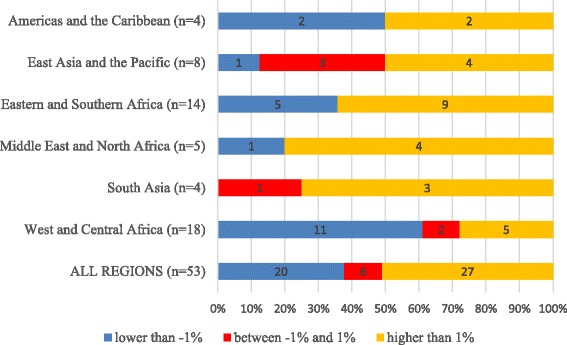
Average exponential growth rate (AEGR) in the number of physicians, nurses and midwives per 10 000 population for 53 Countdown countries with more than one data point between 2004 and the latest available year, by UNICEF region

**Fig. 9:**
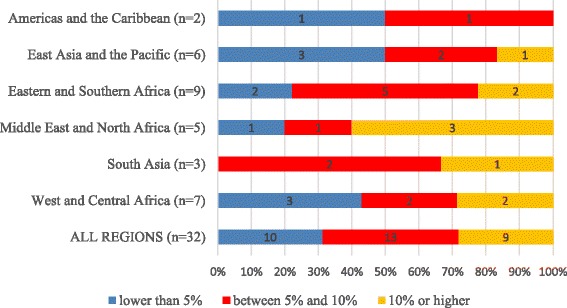
Average exponential growth rate (AEGR) in the number of physicians, nurses and midwives in 32 Countdown countries with positive change between 2004 and the latest available year, by UNICEF region

By UNICEF regions, Fig. 8 shows the direction of change in AEGR and Fig. 9 its magnitude. In all regions except the Americas & Caribbean and West & Central Africa, the number of countries showing a positive AEGR in SHP density was larger than the number showing a negative AEGR (Fig. 8). None of the four South Asian countries recorded a decrease in SHP density, and four of the five countries in Middle East and North Africa recorded an increase. On the other hand, countries in West and Central Africa were the least likely to display an increase in density (only five of 18 countries did). The data from West and Central Africa illustrate well the difficulty of keeping pace with a growing population; 7 out of the 18 countries in this region recorded an increase in SHP *numbers*, but only 5 recorded an increase in SHP *density*.

Figure 9 shows that, in the 32 out of 53 countries (60%) with a positive AEGR in SHP numbers, most (22) recorded an increase of 5% or more, including nine countries with an AEGR of 10% or more. Middle East and North Africa region was a particularly strong performer. By contrast, East Asia and the Pacific and Americas and the Caribbean regions showed weaker growth.

**Fig. 10:**
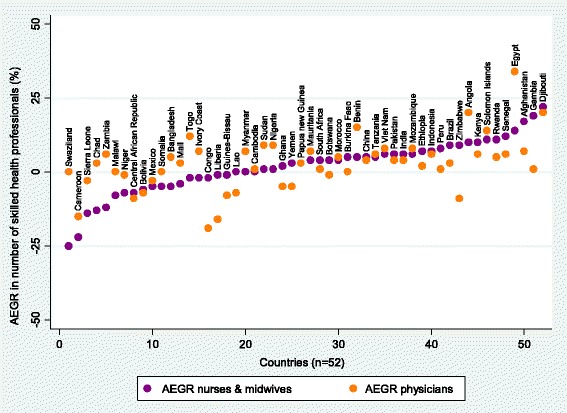
Average exponential growth rate (AEGR) in the number of (a) nurses and midwives and (b) physicians for 52 Countdown countries with positive change between 2004 and the latest available year, and data disaggregated by cadre

For the 52 countries for which it was possible to disaggregate the AEGRs in SHP numbers for physicians and for nurses and midwives, there was a positive correlation between the AEGR in the number of physicians and the AEGR in the number of nurses and midwives (*r* = 0.48, *p* < 0.05). Figure 10 shows the range of values underlying this association. Of 18 countries with a negative AEGR for nurses and midwives, half showed a null or positive AEGR for physicians (Swaziland, Chad, Zambia, Malawi, Somalia, Bangladesh, Mali, Togo and Ivory Coast), i.e. the number of physicians grew while the number of nurses and midwives contracted. The reversed pattern, i.e. a null or positive AEGR for nurses and midwives but a negative one for physicians, was observed in five countries (Lao PDR, Ghana, Yemen, Botswana and Zimbabwe).

**Fig. 11:**
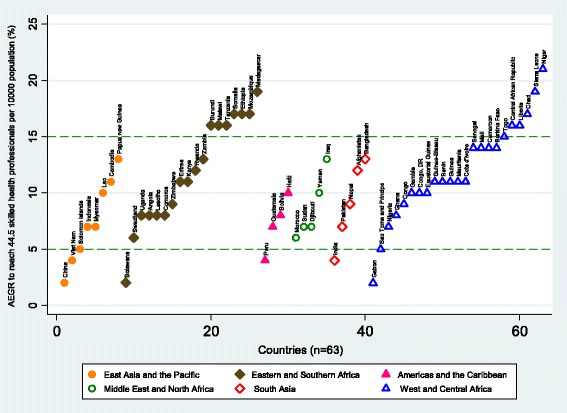
Average exponential growth rate (AEGR) in the number of physicians, nurses and midwives required to reach 44.5 per 10 000 population by 2030 for the 63 Countdown countries currently below this threshold, by UNICEF region

Looking to the future, Fig. 11 shows the required AEGR in SHP numbers that each of the 63 Countdown countries with SHP density below 44.5 SHPs per 10 000 population require to reach that threshold by 2030. Six countries (Botswana, China, Gabon, India, Peru and Viet Nam) require a solid AEGR (<5%) and a further 20 countries (Angola, Bolivia, Comoros, Congo, Djibouti, Ghana, Guatemala, Indonesia, Lesotho, Morocco, Myanmar, Nepal, Nigeria, Pakistan, São Tomé and Principe, Solomon Islands, Sudan, Swaziland, Uganda, Zimbabwe) require a very solid AEGR (5–10%). However, the remaining 37 countries require an extraordinary AEGR of 10% or above, including 13 African countries requiring an unlikely AEGR of 15% or above.

### HRH metrics: a rapid review of the literature

The analysis of the 86 studies included in the rapid review lead to the identification of three different groups, based on the type of methods used (Table 1): (1) basic descriptive and inferential statistics, (2) regression analysis and (3) workforce modelling.

**Table 1:** Number of studies by type of method used and country income group

Type of method	Income level of study setting					Total
	All levels	Low	Lower-middle	Upper-middle	High	
Basic descriptive and inferential statistics	1	17	9	8	13	48
Regression analysis	4	5	6	3	5	23
Workforce modelling	0	2	2	1	10	15
Total	5	24	17	12	28	86

The most frequently used method was basic descriptive and inferential statistics. These studies provided snapshots of the health workforce situation in terms of its availability, geographic distribution [32–48] and other characteristics such as socio-demographic [49–52], deployment and conditions of employment [53–57]. Several studies aiming to characterise migration flows [58–64] and the skill mix of the health workforce [58, 65, 66] also applied this type of method. Others explored planning and health information systems [67–75]. Some focused on measuring inequality [76, 77] (both in terms of socio-economic status and of urban/rural differentials) in access to the health workforce. The results were usually expressed as headcounts by professional cadre and/or as workforce densities.

Regression analysis was the second most common type of method used. The two main types of studies using regression analysis were (1) studies examining the relationship between the availability of health workers and health outcome and health care coverage indicators [27, 78–89] mainly maternal and child outcomes and immunisation rates and (2) studies exploring the relationship between the demographic, socioeconomic and employment characteristics of the workforce and its availability [90–95]. Some studies also focused on workforce planning issues [96–98].

Workforce modelling studies included approaches to predict future health workforce requirements based on different policy scenarios, most often observed in high-income settings. Among the more sophisticated workforce modelling approaches were those based on systems dynamics [99–101] or need for health services [102–104]. Other modelling approaches included supply-based modelling (e.g. focusing on the production and inflows of health workers) [105–108], demand-based modelling (e.g. estimating future health service utilisation) [109] or both [36, 110–112].

Table 2 shows that most studies (and all of the modelling studies) used two or more sources of workforce data. Descriptive studies were most likely to use a combination of primary data collection and analysis of secondary sources, while workforce modelling studies nearly all used secondary analysis only.

**Table 2:** Number and type of sources of data by type of method

Type of method	Basic descriptive and inferential statistics	Regression	Workforce modelling	Total
Number of papers	48	23	15	86
Number of sources used				
1	14 (29%)	7 (30%)	0	21
2	15 (31%)	5 (22%)	4 (27%)	24
3 or more	19 (40%)	11 (48%)	11 (73%)	41
Type of source				
Primary data collection	7 (15%)	4 (17%)	0	11
Analysis of secondary sources	30 (62%)	18 (78%)	14 (93%)	62
Both	11 (23%)	1 (4%)	1 (13%)	13

Out of the 86 studies, 118 data sources were identified: 32 studies combined different types of data sources. National data sources (including professional councils’ registers, ministries of health, national statistics bureaux and national censuses) were the most commonly used data sources across all types of methods. The studies which most often used global data sources (including the WHO Global Health Workforce Statistics Database, WHO health indicator statistics and World Bank socioeconomic indicators) were those using regression analysis. ‘Sub-national’ data sources (administrative databases in, e.g. provinces, districts or health facility registers) were most commonly used in studies using basic descriptive and inferential statistics. Workforce planning studies mostly relied on national-level data sources.

The literature review showed that sophisticated workforce planning approaches are being developed, particularly in high-income settings. These planning approaches look at the population need for health services and align with identified health service priorities. They estimate future health workforce requirements based on populations needs and can be adjusted over time [4, 113, 114]. Such approaches should be considered in all settings, particularly where resources are limited, and are feasible if HRH information systems (HRIS) or national health workforce accounts (NHWA) are already in place [2, 9, 115].

## Discussion

Although this study shows that half of the Countdown countries for which data are available have seen an increase in SHP density and 60% in SHP numbers since 2004, most remain affected by critical needs-based shortages. This situation has hindered the achievement of the MDGs [116], and the fact that so many countries have fewer than the 44.5 SHPs per 10 000 population needed to deliver on the health-related SDGs will negatively affect progress towards these goals. The demand for high, sustained and equitable coverage with proven life-saving interventions will continue to rise especially in sub-Saharan Africa, a challenge compounded by its significant population growth. In many countries, the required scale-up of SHPs may be unrealistic given the resources available and the present capacity of production of qualified health workers.

On the basis of this study and similar analyses [117], it is unlikely that many low- and middle-income countries will be able to address effectively the shortage of health workers without significant additional investments. This will require strategies to mobilise additional resources and funding mechanisms that require long-term strategic planning exercises and a focus on cost-effective primary care delivery models [113]. The increasing diversity in the types of health worker (e.g. by reviewing the scopes of practice of certain cadres, for example expanding the functions of nurses, introducing new cadres such as community-based and mid-level health practitioners, changing the skill mix of cadres placed closer to communities) can be an effective way to make services available, accessible and acceptable and could represent a sustainable strategy to improve health outcomes in some countries. There is also emerging evidence that a more diverse skill mix can represent a cost-effective policy option in low-income settings [118].

Despite the detailed analysis of the HRH, due to data limitations, this study was unable to go beyond descriptive analysis using density as the main HRH measure. The data published in the Global Health Workforce Statistics database are mined from multiple sources and vary within and across countries. The database is largely composed of data from national administrative sources which may be less rigorous on standardised definitions and occupational classifications, as opposed to data collected for differential statistical analysis. It is also noted that national occupation titles and classifications change over time within a country and across countries, posing a challenge to the interpretation of any trend analysis such as the one described here. By and large, administrative sources are confined to the public sector; so the growing private sector in many countries is commonly under- or unrepresented. Hence, there are limitations to HRH data availability and in some instances quality, and far less emphasis on the other dimensions of effective coverage: accessibility, acceptability and quality [119]. Presently, the calculated AEGRs offer the most that can be gleaned from the available data and unfortunately little to no data are reliably and representatively available on the determinants of HRH changes in these countries.

Even if a country has sufficient numbers of health workers, health outcomes will only improve if attention is paid to the other dimensions of effective coverage at sub-national levels because people may be prevented from using services due to geographical, financial or other barriers [120] or may choose not to use services due to concerns over acceptability or quality [121].

HRH data are multi-sectoral and current mechanisms to collect and collate health workforce data routinely do not always include the private and NGO sectors. In many countries, the private sector meets a high proportion of the demand for health services [122], so if excluded, workforce planning is highly compromised and biased. More critically, accurate data on the number of health workers trained using public funds and go on to work in the private sector would enable governments to better manage fiscal resources and the extent of ‘internal brain drain’.

The health-related targets of the SDGs emphasise the need for UHC and therefore equity of access to health care [123]. The lack of standardised, disaggregated and interoperable data on the health workforce limits the capacity of countries to systematically and regularly identify gaps in health worker availability [20, 124], whether these gaps relate to geography, socio-economic group, ethnic group, age, gender or to other variables. Countries cannot address unmet need unless they have reliable information about the nature, size and location of these gaps [113].

The literature review showed that some descriptive studies also focus on the distribution of the health workforce; however, these are often based on cross-sectional data collected from a variety of sources which are not always designed for this specific purpose [125]. Systems for monitoring the health workforce, such as an HRIS embedded in a health management information system (HMIS) [125], should be developed and take into account measures of accessibility, acceptability and quality as well as availability at both national and sub-national levels [9]. It is also important systematically to collect and analyse information on the *dynamics* of the health labour market, such as production, inflows and outflows, distribution, retention and regulation and their determinants. These factors interrelate to impact the availability and quality of health services.

NHWA is a WHO-led initiative which aims “to standardise the health workforce information architecture and interoperability as well as tracking HRH policy performance toward universal health coverage” by defining core indicators and data characteristics [124, 126, 127]. This approach has the potential to break new ground in the standardisation and systematic collection of relevant health workforce information to inform planning and policy development [115]. NHWA build upon existing HRIS in a modular fashion which will be supported by a global digital tool [128]. It is anticipated that, as the implementation of NHWA develops, a standard set of indicators will emerge that will allow more nuanced monitoring of the availability and efficacy of the health workforce [115].

In some countries, concerted investments are needed to professionalise and institutionalise health workforce planning and management. The rapid review shows that innovative approaches are being developed for workforce planning [129] which are less labour-intensive than traditional methods and therefore may represent an opportunity for low- and middle-income countries to introduce systems which are less costly to implement and maintain.

The paper has identified the main sources of HRH data: we recognise that there are limitations in the accuracy and completeness of some of data that can be accessed from these sources and that not all sources have the same strengths and weaknesses as a source of HRH data. In particular, survey-based primary sources and secondary sources may be at risk of being incomplete, out of date or inaccurate due to a lack of full understanding on the part of the researchers, or a focus on ‘what is there’ in terms of data, rather than ‘what is needed’. It should also be noted that in some cases ‘official’ sources may also fall short of complete accuracy, and if these are then used as the basis for input into international databases, then the risk is compounded. International agencies recognise this risk and devote time and effort to clarifying data returns with the original source within the country, but this is not fool-proof. Using a coherent approach to data reporting and analysis based on a template of core indictors as proposed by the implementation of NHWA would gradually eliminate such data errors and shortfalls.

Last but not least, the confinement of reported HRH statistics to SHPs has to be overcome. For example, data are commonly requested on community health workers (CHWs) which have recently been recognised as a cost-effective cadre in delivering certain health services [118], and some countries have chosen to deploy significant numbers of CHWs as a response to the shortage of SHPs, which is not reflected in these analyses. This situation is in part a result of poor reporting of CHW numbers in countries’ official statistics, but mainly due to the lack of standardised definitions of CHWs in terms of training, skills and functions. Until these issues are properly addressed, any global monitoring of those cadres will remain flawed.

The rapid literature review methodology is not as clearly developed as the one established for systematic reviews, so the review may not have been fully comprehensive. In addition, the search terms used are not exhaustive, and the review was conducted by a single researcher, which could have introduced a selection bias. Therefore the findings of the literature review require a careful interpretation.

## Conclusions

The achievement of health-related SDGs remains conditional on the existence of a sufficient health workforce that is well-planned, deployed and appropriately managed and supported to meet population needs. The skill mix, composition and efficiency of such a workforce can only be determined accurately using high-quality and comprehensive data. In many countries, and especially low- and middle-income countries, such data are close to absent. This study adds to the growing body of knowledge on the health workforce trends and on shortcomings of existing health workforce data (see for example, Gupta et al. [130]) by (1) exploring the current need for SHP in maternal and newborn health in Countdown countries in 2015, estimating the necessary growth to meet the HRH requirements to achieve UHC, and (2) highlighting the limitations of the current HRH data sources, HRH metrics and methods of analysis of HRH data. The paper explains the need for a harmonised, global approach to strengthen health workforce knowledge and the evidence base. The Countdown to 2015 collaboration has now evolved into the ‘Countdown to 2030 for Reproductive, Maternal, Newborn, Child, and Adolescent Health and Nutrition’ initiative, which emphasises the need to build, beyond 2015, a solid foundation of baseline data that can be used to track progress and back up the accountability rhetoric with real resources to generate sound data [131], a critical dimension of this relates to the health workforce and the implementation of NHWA.

## Additional file

Additional file 1: Table S1. Initial (either 2004 or closest year before 2004) and latest year with data on SHP numbers in WHO Global Health Workforce Statistics database and most recent estimate of SHP density for 74 Countdown countries. (DOC 86 kb)

## Abbreviations

AEGR: Average exponential growth rate
CHW: Community health worker
GDP: Gross domestic product
GNI: Gross national income
HMIS: Health management information system
HRH: Human resources for health
HRIS: Human resources information system
MDG: Millennium Development Goal
MNH: Maternal and newborn health
NGO: Non-governmental organisation
NHWA: National health workforce accounts
SDG: Sustainable development goal
SHP: Skilled health professional (in this context, physician, nurse or midwife)
UHC: Universal health coverage
UNICEF: United Nations Children’s Fund
WHO: World Health Organization
