# Supplementary material for: Health workforce metrics pre- and post-2015: a stimulus to public policy and planning
Source: Hum Resour Health. 2017 Feb 15;15:14. doi: 10.1186/s12960-017-0190-7 (PMC5312527; doi:10.1186/s12960-017-0190-7)
Supplement: Additional file 1: Table S1. — Initial (either 2004 or closest year before 2004) and latest year with data on SHP numbers in WHO Global Health Workforce Statistics database and most recent estimate of SHP density for 74 Countdown countries. (DOC 86 kb) [file 12960_2017_190_MOESM1_ESM.doc]

**Additional file**

Table: Initial (either 2004 or closest year before 2004) and latest year with data on SHP numbers in WHO Global Health Workforce Statistics database, and most recent estimate of SHP density for 74 Countdown countries

| **Country** | **Initial year** | **Latest year available** | **Skilled health professionals per 10,000 population** |
| --- | --- | --- | --- |
| Afghanistan | 2001 | 2013 | < 22.8 |
| Angola | 2004 | 2009 | < 22.8 |
| Azerbaijan | - | 2013 | ≥ 44.5 |
| Bangladesh | 2004 | 2011 | < 22.8 |
| Benin | 2004 | 2008 | < 22.8 |
| Bolivia | 2001 | 2011 | < 22.8 |
| Botswana | 2004 | 2009 | ≥ 22.8 but < 44.5 |
| Brazil | 2004 | 2013 | ≥ 44.5 |
| Burkina Faso | 2004 | 2010 | < 22.8 |
| Burundi | 2004 | - | < 22.8 |
| Cambodia | 2000 | 2012 | < 22.8 |
| Cameroon | 2004 | 2009 | < 22.8 |
| Central African Republic | 2004 | 2009 | < 22.8 |
| Chad | 2004 | 2006 | < 22.8 |
| China | 2001 | 2011 | ≥ 22.8 but < 44.5 |
| Comoros | 2004 | - | < 22.8 |
| Congo | 2004 | 2007 | < 22.8 |
| Congo, Democratic Republic of | 2004 | - | < 22.8 |
| Cote d’Ivoire | 2004 | 2008 | < 22.8 |
| Djibouti | 2004 | 2008 | < 22.8 |
| Egypt | 2004 | 2009 | ≥ 44.5 |
| Equatorial Guinea | 2004 | - | < 22.8 |
| Eritrea | 2004 | - | < 22.8 |
| Ethiopia | 2004 | 2009 | < 22.8 |
| Gabon | 2004 | - | ≥ 44.5 |
| Gambia | 2004 | 2008 | < 22.8 |
| Ghana | 2004 | 2010 | < 22.8 |
| Guatemala | - | 2009 | < 22.8 |
| Guinea | 2004 | - | < 22.8 |
| Guinea-Bissau | 2004 | 2009 | < 22.8 |
| Haiti | 1998 | - | < 22.8 |
| India | 2004 | 2011 | ≥ 22.8 but < 44.5 |
| Indonesia | 2003 | 2012 | < 22.8 |
| Iraq | - | 2010 | < 22.8 |
| Kenya | 2004 | 2013 | < 22.8 |
| Korea, Democratic People's Republic of | 2003 | - | ≥ 44.5 |
| Kyrgyzstan | - | 2013 | ≥ 44.5 |
| Lao | 2004 | 2012 | < 22.8 |
| Lesotho | 2003 | - | < 22.8 |
| Liberia | 2004 | 2008 | < 22.8 |
| Madagascar | 2004 | 2007 | < 22.8 |
| Malawi | 2004 | 2009 | < 22.8 |
| Mali | 2004 | 2010 | < 22.8 |
| Mauritania | 2004 | 2009 | < 22.8 |
| Mexico | 2004 | 2011 | ≥ 22.8 but < 44.5 |
| Morocco | 2004 | 2009 | < 22.8 |
| Mozambique | 2004 | 2012 | < 22.8 |
| Myanmar | 2004 | 2012 | < 22.8 |
| Nepal | 2004 | - | < 22.8 |
| Niger | 2004 | 2008 | < 22.8 |
| Nigeria | 2003 | 2009 | < 22.8 |
| Pakistan | 2004 | 2010 | < 22.8 |
| Papua New Guinea | 2000 | 2010 | < 22.8 |
| Peru | 1999 | 2012 | ≥ 22.8 but < 44.5 |
| Philippines | 2004 | - | ≥ 44.5 |
| Rwanda | 2004 | 2010 | < 22.8 |
| Sao Tome and Principe | 2004 | - | ≥ 22.8 but < 44.5 |
| Senegal | 2004 | 2008 | < 22.8 |
| Sierra Leone | 2004 | 2010 | < 22.8 |
| Solomon Islands | 2004 | 2009 | ≥ 22.8 but < 44.5 |
| Somalia | 1997 | 2006 | < 22.8 |
| South Africa | 2004 | 2013 | ≥ 44.5 |
| Sudan | 2004 | 2008 | < 22.8 |
| Swaziland | 2004 | 2009 | < 22.8 |
| Tajikistan | - | 2013 | ≥ 44.5 |
| Togo | 2004 | - | < 22.8 |
| Turkmenistan | 2002 | - | ≥ 44.5 |
| Uganda | 2004 | 2005 | < 22.8 |
| Tanzania | 2002 | 2012 | < 22.8 |
| Uzbekistan | - | 2013 | ≥ 44.5 |
| Viet Nam | 2002 | 2013 | ≥ 22.8 but < 44.5 |
| Yemen | 2004 | 2010 | < 22.8 |
| Zambia | 2004 | 2012 | < 22.8 |
| Zimbabwe | 2004 | 2013 | < 22.8 |
